# Pyrotinib combined with thalidomide in advanced non-small-cell lung cancer patients harboring HER2 exon 20 insertions (PRIDE): protocol of an open-label, single-arm phase II trial

**DOI:** 10.1186/s12885-021-08759-8

**Published:** 2021-09-16

**Authors:** Xinghao Ai, Zhengbo Song, Hong Jian, Zhen Zhou, Zhiwei Chen, Yongfeng Yu, Ziming Li, Shun Lu

**Affiliations:** 1 Department of Oncology, Shanghai Lung Cancer Center, Shanghai Chest Hospital, Shanghai Jiao Tong University, No.241 West Huaihai Road, Shanghai, 200030 China; 2 Department of Clinical Trial, The Cancer Hospital of the University of Chinese Academy of Sciences (Zhejiang Cancer Hospital), No.1 East Banshan Road, Hangzhou, 310022 Zhejiang China; 3 Institute of Basic Medicine and Cancer (IBMC), Chinese Academy of Sciences, No.150 Fucheng Road, Hangzhou, 310000 Zhejiang China

**Keywords:** Pyrotinib, Thalidomide, Non-small-cell lung cancer, Human epidermal growth factor receptor 2, Protocol

## Abstract

**Background:**

Standard therapy for human epidermal growth factor receptor 2 (HER2)-mutant non-small-cell lung cancer (NSCLC) is lacking. The clinical benefits with pan-HER inhibitors (afatinib, neratinib, and dacomitinib), anti-HER2 antibody drug conjugate (ADC) trastuzumab emtansine, and an emerging irreversible tyrosine kinase inhibitor (TKI) poziotinib were modest. Another new ADC trastuzumab deruxtecan showed encouraging outcomes, but only phase I study was completed. Pyrotinib, another emerging irreversible epidermal growth factor receptor (EGFR)/HER2 dual TKI, has been approved in *HER2*-positive breast cancer in 2018 in China. It has shown promising antitumor activity against *HER2*-mutant NSCLC in phase II trials, but pyrotinib-related diarrhea remains an issue. The antiangiogenic and immunomodulatory drug thalidomide is a cereblon-based molecular glue that can induce the degradation of the IKAROS family transcription factors IKZF1 and IKZF3. The use of thalidomide can also decrease gastrointestinal toxicity induced by anti-cancer therapy.

**Methods:**

This is an open-label, single-arm phase II trial. A total of 39 advanced NSCLC patients with *HER2* exon 20 insertions and ≤ 2 lines of prior chemotherapy will be recruited, including treatment-naïve patients who refuse chemotherapy. Patients are allowed to have prior therapy with immune checkpoint inhibitors and/or antiangiogenic agents. Those who have prior HER2-targeting therapy or other gene alterations with available targeted drugs are excluded. Eligible patients will receive oral pyrotinib 400 mg once daily and oral thalidomide 200 mg once daily until disease progression or intolerable toxicity. The primary endpoint is objective response rate.

**Discussion:**

The addition of thalidomide to pyrotinib is expected to increase the clinical benefit in advanced NSCLC patients with *HER2* exon 20 insertions, and reduce the incidence of pyrotinib-related diarrhea. We believe thalidomide is the stone that can hit two birds.

**Trial registration:**

ClinicalTrials.gov Identifier: NCT04382300. Registered on May 11, 2020.

## Background

Mutations in human epidermal growth factor receptor 2 (HER2, neu or ERBB2) are found in approximately 2–6.7% of patients with non-small-cell lung cancer (NSCLC) [1–3], and the median overall survival (OS) in this subpopulation is around 22.9 months from the diagnosis of metastatic disease [1]. This mutation is prone to be found in non-smokers and females, and the histological type is more likely to be adenocarcinoma or adenosquamous carcinoma [4–7]. During the past few years, various HER2-targeting therapeutic strategies for *HER2*-mutant NSCLC were developed, including pan-HER inhibitors (afatinib, neratinib, and dacomitinib) [8–10], anti-HER2 antibody drug conjugates (ADCs; such as trastuzumab emtansine [T-DM1]) [11], and emerging irreversible tyrosine kinase inhibitors (TKIs; such as poziotinib) [12]. However, the clinical benefits with these drugs were modest, with the objective response rate (ORR) of 4–44% and median progression-free survival (PFS) of 3.0–5.6 months [8–12]. The new ADC trastuzumab deruxtecan (DS-8201a) brought encouraging clinical benefits for *HER2*-mutant NSCLC. The ORR and median PFS were 73% (8/11) and 11.3 months with trastuzumab deruxtecan in patients with *HER2*-mutant NSCLC, respectively [13]. However, only exploratory phase I results of this novel potent drug were reported at present. Until now, chemotherapy is still the stand of care for this population, and novel treatment strategy is urgently needed.

Pyrotinib is an oral, irreversible epidermal growth factor receptor (EGFR)/HER2 dual TKI. The combination of pyrotinib with capecitabine has been approved for the treatment of patients with *HER2*-positive, relapsed or metastatic breast cancer who were previously treated with taxanes, anthracyclines, and/or trastuzumab in China [14]. In a *HER2* exon 20 insertion patient-derived xenografts model, a more profound tumor regression was observed with pyrotinib than with afatinib and T-DM1 [15]. Two phase II trials have demonstrated the promising antitumor activity and acceptable safety profile of pyrotinib monotherapy in patients with previously treated, *HER2*-mutant advanced NSCLC, with the ORR of 53% (8/15) and 30% (18/60), and median PFS of 6.4 and 6.9 months, respectively [15, 16]. A multicenter, randomized phase III trial (NCT04447118) has been started to compare the efficacy and safety of pyrotinib versus docetaxel in patients with advanced non-squamous NSCLC harboring *HER2* exon 20 mutations who failed platinum-based chemotherapy. However, pyrotinib-related diarrhea remains an issue, with the incidence of 27% (4/15) and 92% (55/60) in previous phase II trials [15, 16]. Thus, we wanted to explore a combination regimen to further enhance the antitumor activity and improve the safety and tolerability of pyrotinib in advanced NSCLC patients with *HER2* exon 20 insertions.

Thalidomide, a derivative of glutamic acid, is an antiangiogenic and immunomodulatory drug. It has been extensively used in patients with multiple myeloma for decades [17, 18]. These years, thalidomide has been proved to be one of the molecular glues that can induce the protein degradation of undruggable targets, which can compromise the limitation of inhibitors [19]. By binding cereblon (CRBN), thalidomide can activate the E3 ubiquitin ligase CRL4^CRBN^-mediated ubiquitination and degradation of the IKAROS family transcription factors IKZF1 and IKZF3 [19], which play key roles in the tumorigenesis and progression of hematologic malignancies [20]. For solid cancers, the clinical activity of thalidomide monotherapy is limited [21], but recent in vitro studies demonstrated the synergistic effects of thalidomide in combination with TKIs on NSCLC [22, 23]. A pilot study of thalidomide plus erlotinib in 52 advanced NSCLC patients with acquired resistance to erlotinib indicated the reversion effect of thalidomide on TKI-acquired resistance [24]. In addition to the antiangiogenic and immunomodulatory actions, the characteristic of thalidomide as a molecular glue may contribute to the synergistic effect in solid cancers. On the other hand, a striking absence of diarrhea was observed when thalidomide was added to chemotherapy [25, 26]. Thus, we hypothesized that the addition of thalidomide to pyrotinib might increase the clinical benefit and reduce the incidence of pyrotinib-related diarrhea.

Therefore, this PRIDE study is conducted to investigate the efficacy and safety of pyrotinib combined with thalidomide in advanced NSCLC patients with *HER2* exon 20 insertions.

## Methods/design

### Study design

**Fig. 1:**
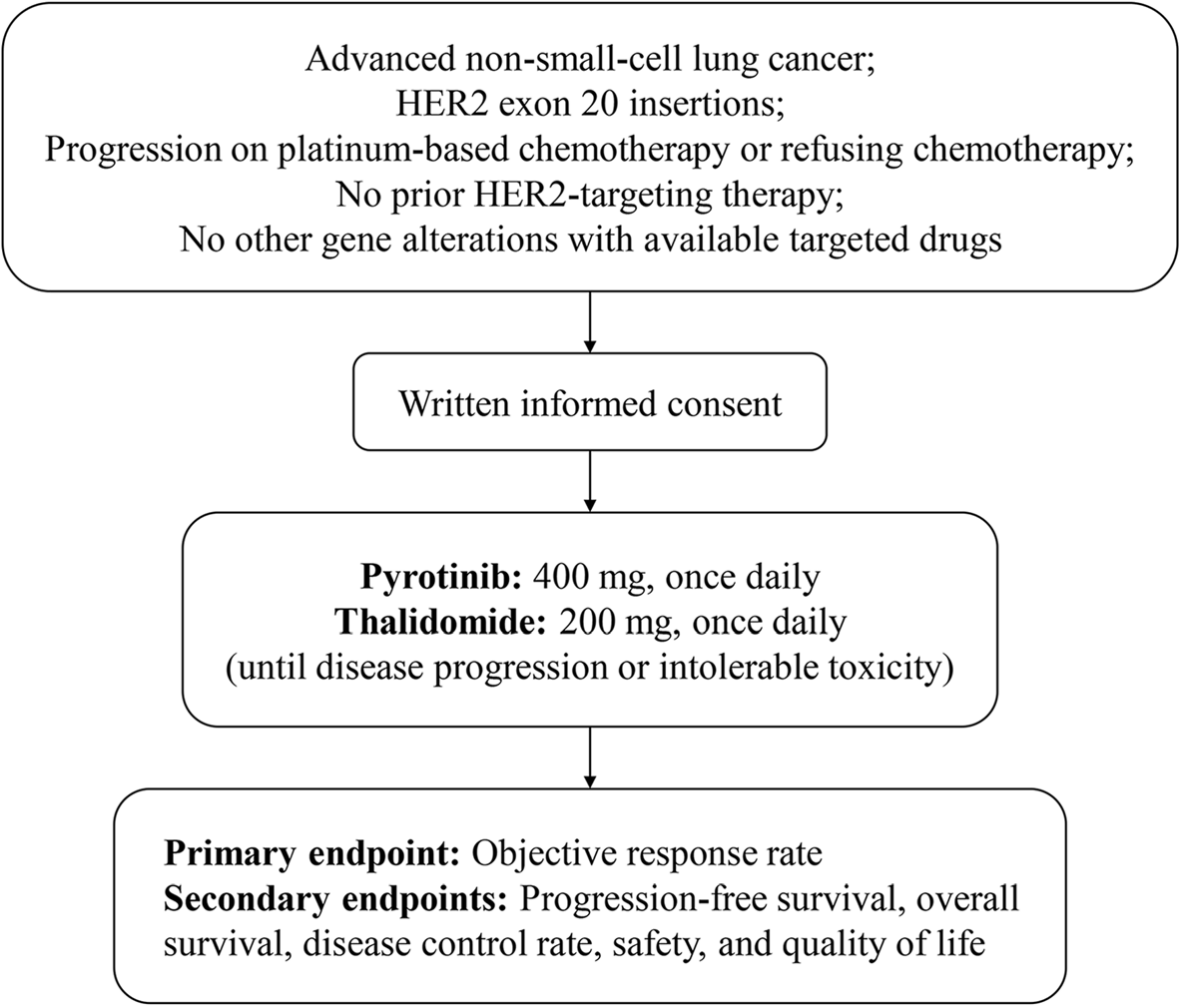
Study design

This is a single-center, open-label, single-arm phase II trial (ClinicalTrials.gov Identifier: NCT04382300) in advanced NSCLC patients with *HER2* exon 20 insertions (Fig. 1). The study is being conducted in accordance with the Declaration of Helsinki and Good Clinical Practice. The protocol and its amendments have been approved by the ethics committee of Shanghai Chest Hospital (No. LS2003). The recruitment was started on May 25, 2020, and the first patient was recruited on June 24, 2020. The enrolment is estimated to be completed in December 2021 for the first stage and in December 2022 for the second stage.

### Eligibility criteria

The patient inclusion and exclusion criteria are detailed in Table 1.

**Table 1:** Eligibility criteria

**Inclusion criteria**	
1	Aged 18–80 years
2	ECOG performance status 0–1
3	Life expectancy ≥3 months
4	At least one measurable lesion according to RECIST 1.1 [27]
5	Histologically or cytologically proved stage IIIB/IV NSCLC according to the 7th edition of TNM classification and staging system for lung cancer published by IASLC [28]
6	*HER2* exon 20 insertions confirmed by next generation sequencing or polymerase chain reaction (if blood samples are used, the mutation abundance should be ≥10%)
7	Disease progression during or after platinum-based chemotherapy, or refusing chemotherapy (patients are allowed to have prior therapy with PD-1/PD-L1 inhibitors and/or antiangiogenic agents)
8	No more than two prior chemotherapy regimens (a. replacing platinum drug due to toxicity is considered as a new regimen; b. adjuvant chemotherapy is not considered as a prior regimen if disease recurrence occurred at more than 6 months after the last dose)
9	No radiotherapy within 3 months, or prior radiotherapy with radiation area < 25% of bone marrow area at least 4 weeks before enrollment
10	Adequate organ function with the following criteria: (a) neutrophil count ≥1.5 × 10^9^/L; platelet count ≥90 × 10^9^/L; Hb ≥90 g/L (b) INR ≤1.5; APTT ≤1.5 × ULN (c) total bilirubin ≤1.5 × ULN; ALT and AST ≤2 × ULN for liver metastases (d) BUN and creatinine ≤1.5 × ULN; creatinine clearance rate ≥ 50 mL/min (e) LVEF ≥50%; QTcF < 470 ms for female and < 450 ms for male patient
11	Willingness to use highly effective contraception from the start of the study to 90 days after the last dose of study drug
12	Written informed consent
**Exclusion criteria**	
1	Prior HER2-targeting therapy before enrollment, including but not limited to trastuzumab, lapatinib, pyrotinib, and neratinib
2	Other gene alterations with available targeted drugs, such as *EGFR* mutations, *T790M* resistance mutations, *ALK* fusions, *ROS1* fusions, *RET* rearrangements, *BRAF V600E* mutations, *NTRK* fusions, and *MET* exon 14 skipping
3	Factors influencing the oral administration of drugs, such as inability to swallow, chronic diarrhea, intestinal obstruction, or other gastrointestinal diseases or abnormalities
4	The third-space effusion, such as pleural effusion, ascites and pericardial effusion, which cannot be controlled by drainage or other methods
5	Radiotherapy, chemotherapy, surgery, or other targeted therapy for non-small-cell lung adenocarcinoma within 4 weeks
6	Active brain metastases, meningeal metastases, spinal compression, or CT or MRI revealing brain or leptomeningeal diseases at screening (patients with symptomatically stable brain metastases can be enrolled if no cerebral hemorrhage is found by brain MRI, CT or venography)
7	Uncontrolled hypokalemia or hypomagnesemia
8	Allergy history to the components of study drug
9	History of immunodeficiency disease (including positive test of human immunodeficiency virus, active hepatitis B/C, or other acquired or congenital immunodeficiency disease) or organ transplantation
10	History of cardiac diseases, including angina, arrhythmia requiring drug therapy or of clinical significance, myocardial infarction, heart failure, and other cardiac diseases unsuitable for this trial as judged by the investigator
11	Thrombotic disease or history of thrombus
12	Other malignancies within 5 years, except for cured cervical cancer in situ, skin basal cell cancer, and skin squamous cell cancer
13	History of neurological or mental disorders, such as epilepsy and dementia
14	Respiratory syndrome (dyspnea ≥grade 2 using NCI CTCAE 5.0)
15	Coagulation disorders (INR > 1.5, prothrombin time > ULN + 4 s, or APTT > 1.5 × ULN), with bleeding tendency or receiving thrombolytic or anticoagulant therapy
16	Renal dysfunction (urine protein ≥++, or 24-h proteinuria ≥1.0 g)
17	Participating in other clinical trials within 4 weeks
18	Pregnant or lactating woman
19	Concomitant diseases seriously affecting the patient safety or the completion of study as judged by the investigator, such as uncontrolled severe hypertension, severe diabetes mellitus, and active infection
20	Any other condition unsuitable for the study as judged by the investigator

*ECOG* Eastern Cooperative Oncology Group, *RECIST* Response Evaluation Criteria In Solid Tumors, *NSCLC* non-small-cell lung cancer, *IASLC* International Association for the Study of Lung Cancer, *PD-1* programmed death-1, *PD-L1* programmed death-ligand 1, *Hb* hemoglobin, *INR* international normalized ratio, *APTT* activated partial thromboplastin time, *ULN* upper limit of normal, *ALT* alanine transaminase, *AST* aspartate transaminase, *BUN* blood urea nitrogen, *LVEF* left ventricular ejection fraction, *QTcF* QT interval corrected by Fridericia’s formula, *CT* computed tomography, *MRI* magnetic resonance imaging, *NCI CTCAE* National Cancer Institute Common Terminology Criteria for Adverse Events

### *HER2* mutation confirmation

*HER2* exon 20 insertions will be confirmed by next generation sequencing, amplification refractory mutation system-polymerase chain reaction, or droplet digital polymerase chain reaction. Tumor tissues obtained from biopsy or circulating tumor DNA from blood samples can be used for HER2 testing. If blood samples are used, the mutation abundance should be ≥10%. The gene mutation reports from other testing organizations are allowed.

### Intervention

Eligible patients will receive oral pyrotinib 400 mg once daily and oral thalidomide 200 mg once daily until disease progression, intolerable toxicity, withdrawal of consent, or other reasons judged by the investigator. Dose adjustment, interruption, or discontinuation of study drug according to the adverse events (AEs) is detailed in Table 2.

**Table 2:** Dose adjustment criteria

NCI CTCAE 5.0	Management (after active treatment and observation)	Dose adjustment after the study drug is continued
**Pyrotinib**		
Clinically significant grade ≥ 2 declined LVEF or LVEF lower than the lower limit of normal (including LVEF declining ≥10% and LVEF < 50% without symptoms, or heart failure)	Discontinue pyrotinib	
Grade 4 diarrhea	Discontinue pyrotinib	
Grade 3 diarrhea or grade 1–2 diarrhea with concomitant symptoms (including but not limited to mild to severe abdominal cramps, grade ≥ 2 nausea or vomiting, declined ECOG performance status, fever, pyemia, decreased neutrophil count, bleeding or dehydration)	Interrupt pyrotinib until diarrhea is restored to grade 0–1 and concomitant symptoms disappear	80 mg reduction each time to the minimum dose of 240 mg
Grade ≥ 2 non-hematologic AEs (except for alopecia, fatigue and asthenia)	Interrupt pyrotinib until diarrhea is restored to grade 0–1	80 mg reduction each time to the minimum dose of 240 mg
Grade ≥ 3 hematologic AEs	Interrupt pyrotinib until diarrhea is restored to grade 0–1	80 mg reduction each time to the minimum dose of 240 mg
**Thalidomide**		
Constipation, somnolence, or peripheral neuropathy	Consider interrupting thalidomide	Dose can be reduced as per investigator’s discretion
Grade 3–4 AEs or clinically significant symptoms	Consider interrupting or discontinuing thalidomide	Dose reduction can be considered
Angioedema, allergic reaction, grade 4 rash, skin peeling, bullae, or any other severe skin reaction	Discontinue thalidomide	

*NCI CTCAE* National Cancer Institute Common Terminology Criteria for Adverse Events, *LVEF* left ventricular ejection fraction, *ECOG* Eastern Cooperative Oncology Group, *AE* adverse event

### Endpoints

The primary endpoint is ORR. The secondary endpoints are PFS, OS, disease control rate, safety, and patient-reported outcomes (European Organization for Research and Treatment of Cancer [EORTC] Quality of Life Questionnaire-Core 30 [QLQ-C30] and Quality of Life Questionnaire-Lung Cancer Module 13 [QLQ-LC13]) [29, 30].

Imaging examinations using computed tomography or magnetic resonance imaging will be conducted at baseline, 3 weeks, and every 6 weeks thereafter. Tumor response will be assessed according to the Response Evaluation Criteria In Solid Tumors, version 1.1 [27]. AEs during the treatment period and within 28 days after the last dose will be recorded and graded according to the National Cancer Institute Common Terminology Criteria for Adverse Events, version 5.0. Survival status will be followed by telephone every 3 months until death, lost to follow-up, or the termination of the study. Subsequent anti-cancer treatment after disease progression or discontinuation of the study treatment will be recorded.

### Statistical analysis

Simon’s two-stage minmax design was selected for sample size calculation. The combination therapy with pyrotinib and thalidomide will be ineffective or uninteresting if the ORR is lower than 30% and this regimen will be worthy of further study if the ORR is ≥50%. A total sample of at least 39 patients is expected to provide 80% power for the analysis at the significance level of 0.05, including 19 patients in the first stage and 20 patients in the second stage of the trial. If six or more patients respond at the completion of the first stage, the second stage can be conducted. Otherwise, the study will be terminated. If at least 16 of 39 patients show response, this combination therapy is effective and warrants further study.

Following intent-to-treat principle, the efficacy analyses will be performed in the full analysis set, defined as all patients who received at least one dose of study drug with at least one efficacy evaluation. Safety analyses will be performed in the safety set, defined as all patients who received at least one dose of study drug with at least one safety record. Descriptive statistics will be conducted on baseline characteristics, tumor response, patient-reported outcomes, and AEs. Survival curves will be plotted using Kaplan-Meier method. No data imputation will be performed for the missing data.

## Discussion

This study will provide evidence on the efficacy and safety of pyrotinib plus thalidomide in advanced NSCLC patients with *HER2* exon 20 insertions, which may be used as a candidate standard therapy.

A previous in vitro study demonstrated the superior effect of pyrotinib on tumor regression compared with afatinib and T-DM1 [15], and indirect comparisons showed a relatively higher median PFS with pyrotinib (6.4–6.9 months) than with other pan-HER inhibitors and ADC T-DM1 (3.0–5.6 months) [8–12]. Although a multicenter, randomized phase III trial (NCT04447118) has been started to verify the efficacy and safety of pyrotinib versus docetaxel in patients with previously treated, advanced non-squamous NSCLC harboring *HER2* exon 20 mutations, we considered that a combination regimen is worth of exploration to further enhance the antitumor activity and improve the safety and tolerability of pyrotinib. We believe thalidomide is the precious stone that can hit two birds.

Patient-reported outcomes are effective tools to directly measure the experiences of patients with cancer, which are more and more important in oncology studies [31]. The incidence of pyrotinib-related diarrhea is terrible, with 92% (55/60) of any grade diarrhea and 20% (12/60) of grade 3–4 diarrhea in a phase II trial [16]. The frequent occurrence of diarrhea negatively impacts patient quality of life and tolerability. Dose adjustment and interruption due to diarrhea may reduce the efficacy of pyrotinib. Constipation caused by thalidomide can counteract the diarrhea from anti-cancer therapy, which is supported by previous clinical trials [25, 26]. In addition, thalidomide can decrease nausea and vomiting induced by chemotherapy [32], which are also the common AEs of pyrotinib [14–16]. We believed that the use of thalidomide with synergistic antitumor effect and attenuation of gastrointestinal toxicity is a better option compared with prophylactic antidiarrheal drugs (such as loperamide) for patients treated with pyrotinib. The use of this combination therapy may improve the tolerability and compliance of patients and do not increase too much financial burden compared with pyrotinib monotherapy, which can be reflected by the results of EORTC QLQ-C30 and QLQ-LC13.

Considering the cardiac toxicity and the risk of thrombosis/embolism events in the use of thalidomide [33, 34], the adequate cardiac function was set as an inclusion criteria and history of cardiac diseases or thrombus were set as exclusion criteria to reduce the risk of patients. However, we still need to be alert to the potential unexpected safety signals when this combination therapy is administered.

In terms of clinical practice, some patients may refuse the chemotherapy due to the tolerability concerns. These patients will be enrolled in the present study, and it should be noted that some patients with previously untreated, advanced NSCLC harboring HER2 exon 20 insertions will receive pyrotinib plus thalidomide. Thus, this study will provide preliminary evidence of this combination therapy in the first-line setting.

We wish that the present study will find a potent chemo-free treatment approach and bring new light of hope for advanced NSCLC patients with *HER2* exon 20 insertions.

## Data Availability

Not applicable.

## Abbreviations

ADC: Antibody drug conjugate
AE: Adverse event
CRBN: Cereblon
EGFR: Epidermal growth factor receptor
EORTC: European Organization for Research and Treatment of Cancer
HER2: Human epidermal growth factor receptor 2
NSCLC: Non-small-cell lung cancer
ORR: Objective response rate
OS: Overall survival
PFS: Progression-free survival
QLQ-C30: Quality of Life Questionnaire-Core 30
QLQ-LC13: Quality of Life Questionnaire-Lung Cancer Module 13
T-DM1: Trastuzumab emtansine
TKI: Tyrosine kinase inhibitor
